# Understanding Fatigue, Insomnia, and COVID-19 PTSS Among Mainland Chinese During Initial Post-Zero-COVID Infection Wave: A Multi-Group Analysis

**DOI:** 10.3390/bs14111033

**Published:** 2024-11-04

**Authors:** Shuo Wang, Yuanyuan Xu, Simon Theodor Jülich, Linman Weng, Qiao Jin, Yuxian Wei, Xu Lei

**Affiliations:** 1 Sleep and NeuroImaging Center, Faculty of Psychology, Southwest University, Chongqing 400715, China; 2 Key Laboratory of Cognition and Personality, Southwest University, Ministry of Education, Chongqing 400715, China; 3 Department of Military Psychology, Army Medical University, Chongqing 400038, China

**Keywords:** insomnia symptoms, fatigue, COVID-19 PTSS, infected and uninfected comparison

## Abstract

In early 2023, China experienced its first widespread COVID-19 outbreak after a policy shift. This study examines the relationship between fatigue and COVID-19-related post-traumatic stress symptoms (PTSS) in infected and uninfected individuals, exploring the potential mediating role of insomnia symptoms. An online survey of 5953 Chinese participants was conducted from 10 to 16 January 2023. Participants reported their COVID-19 infection status, fatigue, insomnia symptoms, and PTSS. Multi-group structural equation modeling (SEM) was used to test whether the mediation paths differed between infected and uninfected groups. The prevalence of fatigue, insomnia symptoms, and COVID-19 PTSS were 30.0%, 36.4%, and 5.8%. The SEM based on the bootstrapping showed that after controlling for demographics, chronic fatigue positively associated with COVID-19 PTSS in a significant way, with insomnia symptoms playing a mediating role. The multi-group analyses further revealed a partial mediation effect of insomnia symptoms on the relationship between fatigue and COVID-19 PTSS in the uninfected group (UG). However, for the infected group (IG), insomnia symptoms fully mediated the relationship between fatigue and COVID-19 PTSS. Infected individuals were more subject to the aforementioned mechanism than uninfected individuals. Addressing chronic fatigue, insomnia, and developing targeted interventions are crucial for supporting mental health across different infection statuses.

## 1. Introduction

### 1.1. The First Wave of Infection After the End of the Zero-COVID Policy in Mainland China

COVID-19 was recognized as a pandemic by the World Health Organization [1], and despite the high financial costs, China implemented strict measures (i.e., mass confinement, social isolation, and increased sanitation) to control the COVID-19 pandemic [2]. These strict measures, also known as a “zero-COVID” policy, were designed to eliminate the virus from a specific geographic area or country rather than simply mitigating its spread. A considerable change happened on 7 December 2022, as ten specifications (referred to as “the 10-point measures”) were officially issued, which started the end of the zero-COVID policy in China [3]. However, the abrupt exit from the zero-COVID policy caused public health concerns about the uncontrolled spread of SARS-CoV-2. Indeed, there are reports showing a sharp rise in the number of infections nationwide [4]. For instance, a national cohort reported that new SARS-CoV-2 infections peaked at 6.36% between 20 and 22 December 2022 [5]. To our knowledge, this was the first wave of infections since mainland China ended its zero-COVID policy. Hospitals in China were overwhelmed by many patients with respiratory diseases [6]. Long-term, China still faces challenges today, such as the health burden of post-COVID-19 conditions. Notably, COVID-19 is still a crisis for everyone, which will continue to have long-term mental health impacts. Therefore, the need arises to understand the mental health status of Chinese residents after the policy changes in order to provide evidence and suggestions for improving people’s mental health in the post-pandemic era.

### 1.2. Fatigue and COVID-19 PTSS

Psychological symptoms and fatigue persist in both the infected and general population during the ongoing COVID-19 pandemic. It was reported that a significant proportion of individuals experienced fatigue regardless of infection status [7,8], showcasing the negative influence of the pandemic on mental health problems. Furthermore, the high risk of infection and the fear of having contracted an infection unknowingly contributed to increased stress-associated psychiatric problems, such as post-traumatic stress symptoms (PTSS) [9]. COVID-19 PTSS are the post-traumatic stress symptoms associated with the COVID-19 pandemic. A pandemic represents a traumatic event that differs in character and spreads from a general traumatic experience. As the nature of trauma has been reconsidered in recent years, it is warranted to examine PTSS in the context of protracted medical emergencies such as epidemics over time [10]. For the general population, research has shown that approximately 13–15% of citizens experienced PTSS even if they were not directly exposed to COVID-19 [11]. Current evidence reveals that pandemic fatigue is significantly associated with PTSS [12]. However, possible mechanisms behind the relationship between fatigue and COVID-19 PTSS remain unclear. Therefore, this study aims to examine the possible mechanisms between fatigue and COVID-19 PTSS.

### 1.3. Insomnia, Fatigue, and COVID-19 PTSS

Insomnia, commonly reported as poor sleep quality, is considered a significant psychological and physiological response to stressful events [13]. As previous studies have shown [14,15], insomnia was highly prevalent during the pandemic. Evidence shows that insomnia symptoms may significantly mediate fatigue and lead to a range of mental health problems [16,17]. Firstly, fatigue increases the risk of insomnia symptoms. A previous study on front-line nurses’ insomnia scores showed that these scores could be predicted by fatigue [18]. Secondly, insomnia symptoms are established risk factors for developing PTSS, although the association between insomnia symptoms and PTSS is complex and bidirectional [19,20]. Insomnia symptoms before analog trauma exposure significantly predict the development of PTSS [21]. Individuals with greater levels of fatigue may experience greater levels of insomnia during the prolonged COVID-19 pandemic, impacting PTSS. Therefore, investigating the potential mediating role of insomnia symptoms in the current study can help to explain how chronic fatigue increases insomnia symptoms, which in turn increases the severity of COVID-19 PTSS.

### 1.4. Group Difference of Infection Status

Previous research comparing sleep and mental health of infected with uninfected people is limited and produced inconsistent findings. Some studies found differences in sleep and mental health problems between infected and uninfected people. For instance, a recent Turkish study showed that the risk of depression symptoms is higher in older adults who were not infected with COVID-19 than in older adults recovering from the disease [22]. An online cross-sectional survey in Pakistan highlighted the poor sleep quality in patients with COVID-19 compared to the uninfected population [23]. However, different previous studies indicated no differences in mental health between infected and uninfected people. For instance, a study in Norway showed that the risk of PTSS of those who had been infected with COVID-19 was not significantly different from that of their non-infected counterparts [24]. In mainland China, the zero-COVID policy recently ended. Therefore, the search for an answer to the question of whether there are differences in sleep and mental health problems between infected and uninfected people (e.g., whether infected people have higher levels or prevalence of PTSS than non-infected people) is of social and medical importance. To approach this goal, it is necessary to compare different underlying mechanisms in the relationship between fatigue and COVID-19 PTSS among infected and uninfected people.

### 1.5. The Present Study

The risk of transmission and death from the COVID-19 infection during the pandemic period created a continuously stressful environment (i.e., worry and depression), increasing the risk of sleep and mental health problems. For instance, a significant proportion of COVID-19-infected and uninfected people reported a number of psychological problems, such as post-traumatic stress symptoms (PTSS), fatigue, and insomnia [12,25,26,27]. Therefore, the current study’s first aim is to examine fatigue, sleep problems, and mental health status of infected and uninfected people in a large sample after the end of the zero-COVID policy.

**Figure 1:**
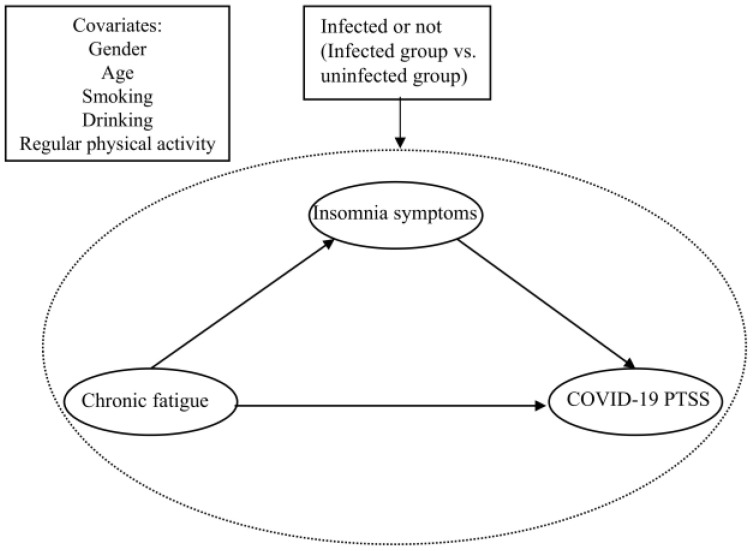
Basic hypothesis model in this study.

With the global pandemic of COVID-19, the differences in psychological responses among individuals from different cultural and social backgrounds have attracted widespread attention. It remains unclear whether there were differences in sleep and mental health problems between infected and uninfected people during the first wave of infections since mainland China ended its zero-COVID policy. Therefore, the second aim of this study is to identify the potential mediating role of insomnia symptoms in the relationship between fatigue and COVID-19 PTSS between these groups during this period. Although 96.05% of the participants in this study were Han Chinese, this cultural background may play an important role in psychological coping strategies and social support acquisition. Understanding the mental health performance of the Han Chinese group during the epidemic is crucial to assessing the impact of cultural differences. Figure 1 presents the conceptual framework. Based on the above review, the following hypotheses are proposed.

**H1.**  *Chronic fatigue is positively related to COVID-19 PTSS.*

**H2.**  *The effect of chronic fatigue on COVID-19 PTSS via insomnia symptoms may differ across UG and IG*.

We hope our results can provide contributions to the psychology of the pandemic and lead to improved psychological guidance for the public and targeted intervention strategies during future epidemics.

## 2. Materials and Methods

### 2.1. Participants and Procedure

We conducted a national internet survey on mental health after the end of zero-COVID using Chinese Netizens as participants. The survey study was conducted following the guidelines of epidemiological observational research. All questionnaires were distributed online via the most commonly used social media platforms in China, such as WeChat, Weibo, and Tencent QQ. Participants who often use social media tools saw this study and completed the survey by scanning the questionnaire address’s Quick Response code (QR code) or clicking the relevant link. Our sampling period was from 10 to 16 January 2023, only one month after China officially issued “the 10-point measures” on 7 December 2022, which ended the zero-COVID policy. The wave of infections that followed occurred very fast, with the number of infected people across the country rising sharply within half a month. During the time of our sampling, most of those infected were still within a month of their infection and had not fully recovered. The period we surveyed was extraordinarily close to the peak of infections [5]. Participants received a mental health report and a randomly determined monetary incentive (1–20 Chinese Yuan) to boost engagement. The local Ethics Committee of Southwest University approved this study. All data collection followed the guidelines of the Declaration of Helsinki.

A total of 7004 participants voluntarily completed the anonymous online questionnaires. All subjects reported their socio-demographic characteristics, COVID-19 infection situation, sleep problems, and mental health problems. To improve the quality of the survey, a series of measures were developed: Allowing each user to submit the questionnaire only once; excluding participants who chose the wrong answer for our quality control question, which instructed respondents to select the sixth option; excluding answers that were deemed as conflicting or illogical in manual control of the questionnaires by two experts (such as answers not conforming to a 24 h time format on the questions regarding bedtime, wake-up time, or actual sleep time). Through these measures, a total of 1051 participants were excluded from this study, leaving 5953 remaining valid respondents (mean age = 26.74 ± 7.25 years) who completed the survey with an effective response rate of 84.99%. All participants gave electronic informed consent before starting this study and were free to withdraw at any moment. Since the survey period was at the end of the zero-COVID policy, we believed the distribution of the infected group (IG) and uninfected group (UG) to be relatively balanced. For the IG and UG, the instructions provided to participants’ questionnaires for collecting COVID-19 information and sleep differed slightly. While IG was asked to report their answers for the period from the day they became infected until the day they filled out the questionnaire, UG was asked to report their situation for the course of the last month.

### 2.2. Measures

#### 2.2.1. Demographic and Health Information

The survey inquired about demographic information, such as age, gender, education level, marital status, monthly family income, smoking, drinking, regular physical activity, history of underlying diseases, and mental disorders. Smoking was defined as consuming one or more cigarettes daily for over six months. Drinking was defined as drinking alcohol twice or more during each week in the six consecutive months prior to the survey. Regular physical activity was defined as more than 20 min of strenuous physical activity at least twice weekly. Previous studies often define regular exercise as more than 30 min of moderate activity five times a week or more than 20 min of strenuous activity three times a week [28]. However, due to the COVID-19 pandemic and related restrictions, we adjusted this definition to more than 20 min of strenuous activity at least twice weekly. This reflects the reduced opportunities for physical activity during the pandemic while still capturing meaningful exercise. Recent COVID-19 infection status information was also collected. COVID-19 infections were reported by subjects subjectively. The IG was also asked to report their current infection situation, time of COVID-19 infection, days from infection to recovery, and severity of their COVID-19 infection (see Supplementary Material Table S5).

#### 2.2.2. Insomnia Symptoms

We assessed insomnia symptoms over the preceding two weeks using the 7-item Insomnia Severity Index (ISI) [29]. Items were scored using a Likert-type scale with 5 response options (0 = not at all to 4 = very much). The total scores range from 0 to 28, with higher scores reflecting more severe insomnia symptoms (0–7 = no clinically significant insomnia; 8–14 = subthreshold insomnia; 15–21 = moderately severe clinical insomnia; 22–28 = severe clinical insomnia). In this study, participants were categorized as having insomnia symptoms if their ISI score was 8 or higher. The Chinese version of ISI is valid and shows good psychometric properties [30]. For this measure, Cronbach’s α was 0.88 in our sample.

#### 2.2.3. Fatigue

The Fatigue Severity Scale (FSS) evaluates fatigue severity and functionality during the past week [31]. The FSS consists of nine items assessed on a 7-point Likert scale ranging from 1 (completely disagree) to 7 (completely agree). The scale evaluates the fatigue experience itself (item 3), the cause of fatigue (item 2), and the effect of that fatigue on the participants’ daily lives (seven items). The average of these nine items is used as a total score, where higher scores demonstrate higher levels of impairment from fatigue. The FSS has shown good reliability and validity in prior assessments [31,32]. An FSS cutoff score ≥ 4 reliably differentiated participants with fatigue from control group participants in prior studies [32]. Therefore, we used a cutoff score ≥ 4 to define fatigue in our study. The Cronbach’s α for this measure in the sample was 0.94.

#### 2.2.4. COVID-19 PTSS

Post-traumatic stress disorder is a persistent mental disorder caused by psychological trauma. The Post-Traumatic Stress Disorder Related to COVID-19 Questionnaire is a self-report questionnaire based on the PTSD Checklist for DSM-5 (PCL-5) to assess specific symptoms concerning the risk of PTSS in an actual pandemic emergency [26]. The original scale, developed in Italy, was modified by deleting two items from the original PCL-5, and adding an extra item related to sleep quality. The focus of the scale lies in the assessment of specific stress-related events during the COVID-19 pandemic, the quarantine measures, and their impact on individual life. This modified scale is hardly comparable to the PCL-5. The scale contains 7 dimensions and 19 items in total, with each response ranging over 5 points from 0 (not at all) to 4 (extremely). The scores of each item are added to obtain the scale’s total score, where a cutoff score of 26 for the scale is considered to correctly categorize participants with PTSS. Considering that the threshold has not been validated in a Chinese sample, this study adopted a different criterion. Scores higher than the mean of the sample plus 1.5 standard deviations were indicative of higher COVID-19 PTSS [33]. Previous research showed good psychometric properties of this scale [34]. Cronbach’s α was 0.97 in the UG sample and 0.96 in the IG sample. The reliability and validity of this measure in our study are further highlighted and explored in the Supplementary Material Tables S1–S4.

### 2.3. Data Analyses

Descriptive statistics, including frequency distributions, were conducted to achieve an overview of socio-demographic variables, the prevalence of fatigue, insomnia symptoms, and COVID-19 PTSS. We used chi-square tests to compare the prevalence of COVID-19 PTSS among the groups. Pearson’s correlations were used to perform correlation analysis. Structural equation modeling (SEM) with the maximum likelihood method was performed with AMOS 23.0 to test the hypothetical model. First, a measurement model was carried out using confirmatory factor analysis (CFA). Then, the direct and indirect effects of chronic fatigue were tested using the bootstrapping estimate. Bootstrapping (5000 bootstrap samples) with 95% confidence intervals (CIs) was conducted to calculate the significance of indirect effects. The 95% CIs did not include zero, indicating significant effects. A *p*-value less than 0.05 was considered statistically significant. In addition, multi-group SEM was used to evaluate whether the proposed mediation model showed significant differences between uninfected and infected groups. The critical difference ratio (CRD) was used to determine the inter-path comparison to check for significant differences in each structural path in the two groups. When the absolute value of CRD was greater than 1.965, there was a difference between groups at the level of *p* < 0.05. Considering that the Chi-square (χ^2^) is sensitive to sample size, the following criteria were used to evaluate fit indicators: comparative fit index (CFI) > 0.90, Tucker-Lewis fit index (TLI) > 0.90, root mean square error of approximation (RMSEA) < 0.08 (Wen et al., 2004) [35]. All data were analyzed using IBM SPSS Statistics 25.0 and AMOS 23.0.

## 3. Results

### 3.1. Demographic Information

Demographic information and sample differences related to COVID-19 infections are presented in Table 1. Of all participants included in the sample, the majority were male (67.0%), unmarried (60.7%), well educated (81.1% ≥ bachelor’s degree), had no history of underlying diseases (77.6%), and had no history of mental disorders (76.8%). The sample was located in diverse geographical locations inside China, including 31 provinces, municipalities, and autonomous regions. For instance, subjects participated from Tianjin (16.66%), Beijing (16.27%), Hebei (15.08%), Chongqing (6.68%), and other regions. Of all participants, more than half were between 18 and 29 years old. Most participants had a monthly family income of <20,000 yuan. In the sample of 5953 participants, 39.2% were classified as UG (*n* = 2336), and 60.8% were classified as IG (*n* = 3617).

### 3.2. The Prevalence of Fatigue, Insomnia Symptoms, and COVID-19 PTSS

The overall prevalence of fatigue, insomnia symptoms, and COVID-19 PTSS found are 30.0%, 36.4%, and 5.8%, respectively. For the UG, the prevalence of fatigue, insomnia symptoms, and COVID-19 PTSS found are 19.3%, 29.1%, and 8.0%. For the IG, the prevalence of fatigue, insomnia symptoms, and COVID-19 PTSS found are 36.9%, 41.0%, and 4.4%. The proportion of COVID-19 PTSS found in the UG is higher than that found in the IG (8.0% vs. 4.4%, *p* < 0.05).

### 3.3. The Relationships Between Fatigue and Insomnia or COVID-19 PTSS

Pearson’s correlations are presented in Table 2. As expected in both the IG and the UG, fatigue and insomnia symptoms are positively correlated with COVID-19 PTSS (*r* = 0.15–0.41, all *p*-values < 0.01). Insomnia symptoms were most significantly correlated with PTSS in both groups (UG: 0.38, IG: 0.41).

### 3.4. Test of the Measurement Model

The measurement model was tested using confirmatory factor analysis (CFA). The results showed acceptable model fit indices: (CFI = 0.951, TLI = 0.945, RMSEA = 0.067). All observed variables were significantly loaded on the corresponding underlying constructs. The factors of the three latent variables (chronic fatigue, insomnia symptoms, and PTSS) were 0.652–866, 0.627–0.759, and 0.891–0.950, respectively. These results show that the latent variables chosen represent the corresponding observed variables well.

### 3.5. Test of the Structural Model

**Figure 2:**
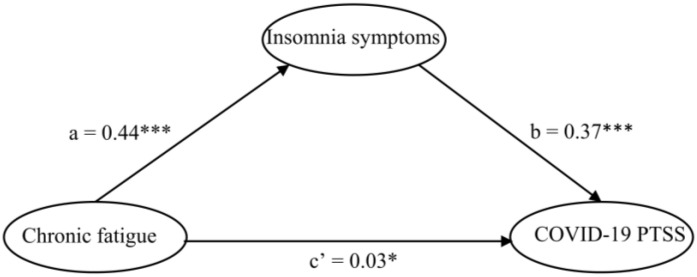
Standardized structural mode in full sample. * *p* < 0.05, ***: *p* < 0.001.

The results of the structural model revealed good statistics fit: χ^2^ = 6755.663, *df* = 327, *p* < 0.001, CFI = 0.950, TLI = 0.942, RMSEA = 0.058. The hypothesized structural model included chronic fatigue, insomnia symptoms, and PTSS as endogenous variables (see Figure 2). As shown in Figure 2 and Table 3, in the full sample model after controlling for age, gender, drinking, smoking, and regular physical activity, chronic fatigue positively predicted COVID-19 PTSS (*β* = 0.03, *p* = 0.0499 < 0.05) and insomnia symptoms positively predicted COVID-19 PTSS (*β* = 0.37, *p* < 0.001). Moreover, chronic fatigue negatively predicted insomnia symptoms (*β* = 0.44, *p* < 0.001). As shown in Table 4, the bootstrap 95% CI remained above zero, indicating that insomnia symptoms partially mediated the relationship between fatigue and COVID-19 PTSS (B = 0.430, 95% bootstrap CI [0.386, 0.474]). This accounted for 85.49% of the total effect.

### 3.6. Test of Group Differences

Group differences were determined using multi-group analysis in SEM. First, the invariance of the measurement model was tested. According to the fitting criteria of the measurement invariance model [36], compliance with invariance is indicated when the changes in the CFI (ΔCFI) and the RMSEA (ΔRMSEA) are less than 0.010. The results showed that the increase of CFI and RMSEA values was less than 0.010, supporting measurement invariance across the samples. Second, unconstrained structural models that allow structural paths to vary with infection status are compared with constrained structural models that constrain the equality of factor loads, covariance, weights, and residuals between UG and IG. The results showed that the differences between the unconstrained model (χ^2^ = 117.46, *df* = 20) and the constrained model (χ^2^ = 213.21, *df* = 33) are significant (*p* < 0.001).

The critical ratios of differences (CRD) test showed significant divergences across UG and IG in the two pathways. Firstly, the structural path from chronic fatigue to insomnia symptoms was significantly different across UG and IG (CRD = 5.921, *p* < 0.05); secondly, it showed significant divergences between UG and IG in the path coefficient of chronic fatigue to COVID-19 PTSS (CRD = 3.793, *p* < 0.05).

**Figure 3:**
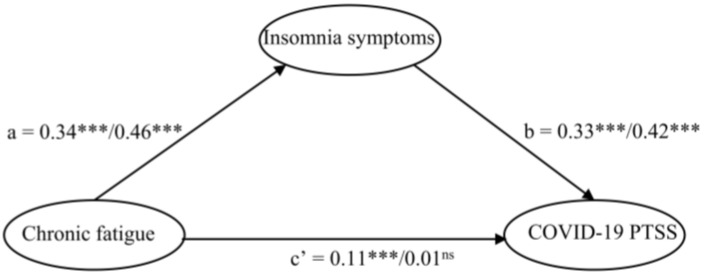
Standardized structural mode in uninfected/infected group. The paths in both yield significant differences between the two groups. ***: *p* < 0.001, ns = non-significant.

As shown in Figure 3 and Table 3, two structural paths had significant effects on IG and UG, but the degree of effect varied. Specifically in the path from chronic fatigue to COVID-19 PTSS, the effect on the UG (*β* = 0.11, *p* < 0.001) was more significant than that on the IG (*β* = 0.01, *p* > 0.05). On the path connecting chronic fatigue to insomnia symptoms, the UG (*β* = 0.34, *p* < 0.001) was less affected than the IG (*β* = 0.46, *p* < 0.001). Although no significant group differences were found between UG and IG in the path from insomnia symptoms to COVID-19 PTSS, the effect of chronic fatigue on COVID-19 PTSS was weaker in the UG compared to the IG (UG: *β* = 0.33; IG: *β* = 0.42). As shown in Table 4, The results of bootstrap showed that chronic fatigue had significant indirect effects on COVID-19 PTSS via insomnia symptoms in the UG (95% bootstrap CI [0.172, 0.461]). However, the mediating effect was not significant in the IG (95% bootstrap CI [−0.076, 0.131]).

Finally, we conducted additional factor analyses to specifically address the issue of overlapping symptom items and their contribution to the variance in our study’s outcomes. Specifically, the two items of COVID-19 PTSS (“to have difficulty falling asleep,” and “to have a disturbed sleep”) were deleted in the additional analysis. This additional analysis revealed that the overlap of these symptom items did not significantly alter the reported variance or the conclusions drawn from our study (see Supplementary Material Tables S7 and S8).

## 4. Discussion

This study is the first of its kind to evaluate mental health problems while differentiating between infected and uninfected people in the first wave of widespread COVID-19 infections in China. The average COVID-19 PTSS prevalence was 5.8% in the sample. The prevalence of COVID-19 PTSS was 8.0% in the UG and 4.4% in the IG. Additionally, the multi-group analyses confirmed the different mediating effects of insomnia symptoms on the relationship between fatigue and COVID-19 PTSS in the IG and the UG. We found a partial mediation effect of insomnia symptoms on the relationship between fatigue and COVID-19 PTSS in the uninfected group (UG). However, in the infected group (IG), we found that insomnia symptoms fully mediated the relationship between fatigue and COVID-19 PTSS.

### 4.1. High Risk of Fatigue, Insomnia, and COVID-19 PTSS

In this study, we found prevalences of fatigue, insomnia symptoms, and COVID-19 PTSS in subjects of 30.0%, 36.4%, and 5.8%, respectively, indicating that the uncertainty of the epidemic development caused more significant psychological pressure on the public after the end of the zero-COVID policy. The sudden policy shift may have caused some “maladaptive reactions”. For example, the fear of infection in the face of a surge of infections and the fear of not knowing when the epidemic will end can amplify psychological distress and produce severe sleep problems and psychological symptoms as seen in the early and later stages of the COVID-19 pandemic [37,38]. The prevalence of fatigue we found in this study was slightly lower than what was found in previous studies during the COVID-19 pandemic, both in the general population (such as college students) and in specific populations (such as nurses or infected people) [39,40,41]. Individuals are unprepared for sudden changes in epidemic prevention and control policies, creating concerns about infection, virus mutation, and the resurgence of new coronary pneumonia. This unpreparedness results in insufficient physical and mental self-care ability and increased fatigue. The prevalence of insomnia symptoms in our subjects was 36.4%, which is lower than the prevalence of insomnia found during the initial first wave of the coronavirus epidemic [37]. The reduction in the prevalence of insomnia after the deregulation could reflect a combination of factors such as a return to normal life, a reduction in social and psychological stress, and a reduction in people’s concern about the epidemic.

Interestingly, the prevalence of COVID-19 PTSS found in this study (5.8%) seems to be higher than that found in previous studies [38], which may be caused by the fact that our data were collected during the peak period of infections after the policy shifts characterized by a high degree of overload in national health systems, i.e., a phase that can be perceived as a very acute stressor in the Chinese public. Specifically, the rapid changes, uncertainty, reoccurrences of traumatic experiences, and continued anxiety about the pandemic that China experienced after the sudden liberalization of controls following the end of the zero-COVID policy could have increased the risk of PTSS. Another possible explanation could be that the COVID-19 PTSD questionnaire used in this study focuses on assessing the specific stress-related events of the COVID-19 pandemic, which could have caused participants to report more PTSS for this particular period. Our findings highlight the importance of considering mental health interventions and support in health public events.

Notably, compared with the UG, we found a higher prevalence of fatigue and insomnia symptoms in the IG. The experience of COVID-19 can trigger significant psychological distress. Anxiety about the potential long-term effects of the virus, worries about passing it on to others, and stress during isolation may lead to sleep disturbances and fatigue. Especially participants in the UG had a higher COVID-19 PTSS prevalence than those in the IG, which is inconsistent with previous results of Chinese studies [42]. Possible explanations are as follows: Individuals who were infected and recovered may have developed a mindset of “having experienced the worst” because they directly faced and overcame the disease, which may have reduced their fear of possible future threats to a certain extent. At the same time, uninfected individuals may still hold elevated concerns and fears about potential infection risks [43]. The Supplementary Material Table S6 also supports this, as uninfected individuals scored higher on measures of anxiety, depression, stress, and loneliness compared to those who were infected. These factors can magnify mental stress and explain the increased risk for COVID-19 PTSS of the UG compared to that of the IG.

### 4.2. Different Relationships Between Age, Regular Physical Activity, and COVID-19 PTSS Across UG and IG

The results showed that age was negatively and statistically significantly correlated with COVID-19 PTSS scores, but only in the IG. Similarly, a survey of a US sample showed that COVID-19 worry scores were negatively correlated with age [44]. In other countries, there was also an inverse relationship between age and COVID-19-related concerns in the early stages of the pandemic [45]. This may suggest an acquired psychological resilience with age or a different perception of risk and mortality. Older individuals may have developed greater psychological resilience due to life experiences [46], enabling them to better manage emotional distress in the face of COVID-19. Age-related psychological resilience may help mitigate the psychological impacts of the infection, leading to lower PTSS scores in older adults.

Our findings showed that regular physical activity was negatively and statistically significantly correlated with COVID-19 PTSS scores, but only in the IG. This may suggest that in those who were infected, physical activity could have played a crucial role in both physical and psychological recovery. Specially, physical activity can boost immune function, which may have aided in the recovery from infection [47]. Additionally, physical activity helps reduce psychological stress and anxiety, which are often heightened during and after infection. For instance, previous research has shown that regular adherence to physical activity guidelines is strongly linked to a decreased risk for serious outcomes in adults with COVID-19 [48]. A study also supported the idea that regular physical activity can reduce the risk of SARS-CoV-2 infection [49]. At the same time, in UG, regular physical activity was not significantly associated with COVID-19 PTSS. This may be because uninfected people may face less direct physical and psychological stress, which may undermine the link between physical activity and PTSS. However, this is only our preliminary interpretation of the observed correlation. To further understand this relationship, future studies will need longitudinal designs to examine the long-term association between regular physical activity and PTSS.

### 4.3. Different Mediating Effects of Insomnia Between Fatigue and COVID-19 PTSS Across UG and IG

The results of this study show that insomnia symptoms mediate the relationship between chronic fatigue and COVID-19 PTSS in the full sample. The overall sample analysis shows a significant direct effect, while the Bootstrap confidence intervals include zero, possibly due to the variability in mediation patterns across different groups. In one group we found a partial mediation, while in the other we found a full mediation, suggesting that the overall effect might be diluted or masked when aggregating the data. The finding underscores the importance of conducting group-specific analyses in mediation models. Specifically, regardless of infection status, insomnia partially mediates the relationship between fatigue and COVID-19 PTSS. This finding is similar to previous studies, showing that insomnia mediates the relationship between stress and PTSS [16]. COVID-19 can be defined as a traumatic experience with consequences of exposure to severe psychological stress. According to the “3P” model of insomnia, the precipitating factor of stress accelerates the development of acute insomnia [50]. Based on the trauma insomnia model, trauma disrupts normal sleep–wake regulatory mechanisms by sensitizing the arousal center of the central nervous system [51]. Disturbed sleep can in turn prevent people with PTSS from fully erasing fear-based memories, impair coping abilities, and disrupt emotional regulation. All of this may exacerbate the severity of PTSS [52,53]. Therefore, the end of the zero-COVID policy after the pandemic as a stressful life event may trigger the onset and maintenance of insomnia.

In the UG of our sample, fatigue is significantly associated with PTSD symptoms; however, this association was not significant in the IG. Similarly, a recent study in South Korea showed that pandemic fatigue was significantly associated with post-traumatic stress symptoms among general college students [12]. Among infected persons, chronic fatigue is likely to exacerbate PTSS by triggering insomnia symptoms. A larger percentage of the infected group screened positive for insomnia symptoms; therefore, a logical conclusion would be that these simply overwhelm the effects of fatigue in that group. Among uninfected participants, the direct effects of chronic fatigue on PTSS turned out to be more significant than those of insomnia symptoms, although they also played a mediating role. A possible explanation for this is that the items of the COVID-19 PTSD questionnaire we used measured direct stressors (i.e., fear of the infection) and indirect stressors (i.e., social distancing) consequent to the COVID-19 emergency. Uninfected people showed more psychological distress about the fear of being infected than those infected. Therefore, uninfected individuals are more affected by fatigue due to the COVID-19 pandemic and may be more susceptible to PTSS because of the taxing effects on the psychological and physical health of voluntary restrictions on activity to reduce exposure and transmission of the virus.

To alleviate the psychological stress of the public, the follow-up psychological intervention should focus on strengthening the public’s psychological health via counseling and emotional comfort. For the uninfected population, more emphasis should be placed on providing chronic fatigue management strategies, including nutritional support, moderate exercise, psychological counseling, etc., in order to directly reduce fatigue and therefore the direct impact on PTSS, while for the infected population, the primary focus should be placed on the relief of insomnia symptoms. Providing Cognitive Behavioral Therapy for Insomnia (CBT-I) and educating on sleep hygiene to reduce PTSS could be effective measures too. These measures could provide the general population with better sleep to boost immunity and thus reduce stress and COVID-19 PTSS.

### 4.4. Limitations

There are some limitations to this study. First, due to the cross-sectional study design, causal relationships cannot be inferred, and it remains unclear to what extent PTSS (and insomnia) may be attributed to factors from earlier phases of the pandemic (e.g., centralized isolation). Future studies should confirm the causal links and assess the cumulative impact of pandemic stressors on mental health. Second, data collection relied on online self-reports, which could have produced potentially biased results due to sample selection and limited generalizability. Future research should employ more reliable and valid methods beyond online surveys. Third, our measure of insomnia, daytime sleepiness, and COVID-19 PTSS by self-reported questionnaire consisted only of screening assessments, and further clinical diagnosis of mental illness needs to be conducted. Fourth, a limitation of this study is the difference in the reporting periods between the infected group (IG) and the uninfected group (UG). This variation may introduce biases, such as measurement bias, recall bias, and comparison bias. IG participants may recall their experiences more vividly due to the recency of the infection, whereas UG participants, recalling over a longer period, may provide less accurate reports. Future studies should aim to standardize reporting periods across groups to minimize these biases and improve data comparability. Finally, the grouping of IG and UG participants in this study is not based on clinical diagnosis, which may be an underestimation of the infected population and an overestimation of the uninfected population due to the neglect of some asymptomatic infected participants.

## 5. Conclusions

This study found a prevalence of fatigue, insomnia symptoms, and COVID-19 PTSS among the sample of both infected and uninfected people in China after the end of the zero-COVID policy. Furthermore, we found different mediation effects of insomnia on the relationship between fatigue and COVID-19 PTSS in uninfected and infected individuals. This study found that most of the participants were Han Chinese. This cultural background may have affected their psychological responses to the epidemic. Future research should still consider ethnic minority groups from different cultural backgrounds to gain a more comprehensive understanding of how cultural differences affect mental health. In summary, as the world continues to address COVID-19 and other health crises, the need for adaptable, targeted, and culturally sensitive mental health services becomes increasingly clear.

## Figures and Tables

**Table 1:** Population characteristics of this study cohort in relation to COVID-19 infection. (*n* = 5953).

Characteristics	Total Number of Participants	Uninfected Group	Infected Group	χ^2^/*t*	*p*-Value
Infection status					
Uninfected	2336 (39.2%)				
Infected	3617 (60.8%)				
Age (years) (Mean ± SD)	26.74 ± 7.25	26.52 ± 7.00	26.88 ± 7.39	6.86	0.077
<18	70 (1.2%)	32 (1.4%)	38 (1.1%)		
18–29	3827 (64.3%)	1450 (62.1%)	2377 (65.7%)		
30–39	1081 (18.2%)	422 (18.1%)	659 (18.2%)		
40–49	244 (4.1%)	81 (3.5%)	163 (4.5%)		
50–59	56 (0.9%)	12 (0.5%)	44 (1.2%)		
≥60	18 (0.3%)	9 (0.4%)	9 (0.2%)		
Gender (*n*, %)				23.27	<0.001
Females	1966 (33.0%)	686 (29.4%)	1280 (35.4%)		
Males	3987 (67.0%)	1650 (70.6%)	2337 (64.6%)		
Marital status (*n*, %)				9.58	0.022
Married	2202 (37.0%)	821 (35.1%)	1381 (38.2%) *		
Unmarried	3615 (60.7%)	1453 (62.2%)	2162 (59.8%)		
Divorced	111 (1.9%)	54 (2.3%)	57 (1.6%) *		
Widowed	25 (0.4%)	8 (0.3%)	17 (0.5%)		
Education level (*n*, %)				35.56	<0.001
Until middle school	143 (2.4%)	81 (3.5%)	62 (1.7%) *		
Technical secondary school or high school	898 (15.1%)	401 (17.2%)	497 (13.7%) *		
College	3786 (63.6%)	1421 (60.8%)	2365 (65.4%) *		
Master	947 (15.9%)	357 (15.3%)	590 (16.3%)		
Doctor’s degree or above	179 (3.0%)	76 (3.3%)	103 (2.8%)		
Monthly family income (*n*, %)				21.24	0.001
<1000 CNY	117 (2.0%)	61 (2.6%)	56 (1.5%) *		
1000 CNY-5000 CNY	854 (14.3%)	327 (14.0%)	527 (14.6%)		
5000 CNY–10,000 CNY	2226 (37.4%)	892 (38.2%)	1334 (36.9%)		
10,000 CNY–20,000 CNY	1845 (31.0%)	679 (29.1%)	1166 (32.2%) *		
20,000 CNY–50,000 CNY	650 (10.9%)	253 (10.8%)	397 (11.0%)		
>50,000 CNY	261 (4.4%)	124 (5.3%)	137 (3.8%) *		
Smoking (*n*, %)				4.38	0.036
No	3822 (64.2%)	1462 (62.6%)	2360 (65.2%)		
Yes	2131 (35.8%)	874 (37.4%)	1257 (34.8%)		
Drinking (*n*, %)				10.19	0.001
No	4102 (68.9%)	1554 (66.5%)	2548 (70.4%)		
Yes	1851 (31.1%)	782 (33.5%)	1069 (29.6%)		
Regular Physical activity (*n*, %)				9.99	0.002
No	3682 (61.9%)	1387 (59.4%)	2295 (63.5%)		
Yes	2271 (38.1%)	949 (40.6%)	1322 (36.5%)		
History of underlying diseases (*n*, %)				32.60	<0.001
No	4617 (77.6%)	1722 (73.7%)	2895 (80.0%)		
Yes	1336 (22.4%)	614 (26.3%)	722 (20.0%)		
History of mental disorders (*n*, %)				11.65	0.001
No	4570 (76.8%)	1739 (74.4%)	2831 (78.3%)		
Yes	1383 (23.2%)	597 (25.6%)	786 (21.7%)		
Insomnia symptoms (*n*, %)				87.15	<0.001
ISI < 8	3789 (63.6%)	1650 (70.9%)	2133 (59.0%)		
ISI ≥ 8	2164 (36.4%)	680 (29.1%)	1484 (41.0%)		
Fatigue (*n*, %)				207.15	<0.001
FSS ≤ 4	4168 (70.0%)	1884 (80.7%)	2284 (63.1%)		
FSS > 4	1785 (30.0%)	452 (19.3%)	1333 (36.9%)		
COVID-19 PTSS (*n*, %)				33.07	<0.001
COVID-19 PTSS < (Mean + 1.5 × SD)	5608 (94.2%)	2150 (92.0%)	3458 (95.6%)		
COVID-19 PTSS ≥ (Mean + 1.5 × SD)	345 (5.8%)	186 (8.0%)	159 (4.4%)		

FSS: Fatigue Severity Scale; ISI: Insomnia Severity Index; COVID-19 PTSS: Post-traumatic stress symptoms related to the COVID-19 pandemic; SD: standard deviations; *: *p* < 0.05.

**Table 2:** Correlations between the main study variables and COVID-19 PTSS.

Variables	COVID-19 PTSS Scores	
	UG	IG
Gender	0.09 **	0.16 **
Age	−0.01	−0.11 **
Smoking	0.22 **	0.26 **
Drinking	0.21 **	0.26 **
Regular physical activity	0.01	−0.11 **
Fatigue	0.22 **	0.15 **
Insomnia symptoms	0.38 **	0.41 **

COVID-19 PTSS: Post-traumatic stress symptoms related to the COVID-19 pandemic. UG: uninfected group. IG: infected group. ** *p* < 0.01.

**Table 3:** Results of structural model for full sample and subsamples.

Model Paths	Full Sample			UG			IG		
	*β*	SE	CR	*β*	SE	CR	*β*	SE	CR
COVID-19 PTSS <--- Chronic fatigue	0.03 *	0.01	30.423	0.11 ***	0.06	5.080	0.01	0.05	0.537
COVID-19 PTSS <--- Insomnia symptoms	0.37 ***	0.09	24.374	0.33 ***	0.16	14.303	0.42 ***	0.10	21.603
Insomnia symptoms <--- Chronic fatigue	0.44 ***	0.04	1.961	0.34 ***	0.01	14.668	0.46 ***	0.01	24.978

*** *p* < 0.001, * *p* < 0.05; UG: uninfected group; IG: infected group; *β*, standardized coefficient; SE: standard error; CR: critical ratio; COVID-19 PTSS: Post-traumatic stress symptoms related to the COVID-19 pandemic; adjusted for age, gender, drinking, smoking, and regular physical activity.

**Table 4:** Direct and indirect effects and 95% confidence intervals (CI).

Model Pathways	Full Sample			UG			IG		
	Estimated	95%CI		Estimated	95%CI		Estimated	95%CI	
		Lower	Upper		Lower	Upper		Lower	Upper
Direct effect									
COVID-19 PTSS <--- Chronic fatigue	0.07	−0.121	0.158	0.32	0.172	0.461	0.02	−0.076	0.131
Indirect effect									
COVID-19 PTSS <--- Insomnia symptoms <--- Chronic fatigue	0.43	0.386	0.474	0.33	0.278	0.403	0.49	0.437	0.556

UG: uninfected group; IG: infected group; CI: confidence intervals.

## Data Availability

The data of this manuscript will not be deposited. The datasets generated and/or analyzed during this study are not publicly available. The data that support the findings of this study were collected by the researcher and were strictly used for research purposes in this study.

## Supplementary Materials

The following supporting information can be downloaded at: https://www.mdpi.com/article/10.3390/bs14111033/s1. Table S1. Descriptive statistics of the 19 items of COVID-19 PTSD in infected group; Table S2. Descriptive statistics of the 19 items of COVID-19-PTSD in uninfected group. Table S3. Comparison of the scores of items in the high and low groups of COVID-19 PTSD among infected group Table S4. Comparison of the scores of items in the high and low groups of COVID-19 PTSD among uninfected group. Table S5. Specific information on COVID-19 infection and recovery of infected groups (*n* = 3617). Table S6. Comparison of emotional symptoms among uninfected group and infected group. Table S7. Results of structural model for full sample and subsamples. Table S8. Direct and indirect effects and 95% confidence intervals (CI).
