# Supplementary material for: Understanding Fatigue, Insomnia, and COVID-19 PTSS Among Mainland Chinese During Initial Post-Zero-COVID Infection Wave: A Multi-Group Analysis
Source: Behav Sci (Basel). 2024 Nov 4;14(11):1033. doi: 10.3390/bs14111033 (PMC11590877; doi:10.3390/bs14111033)
Supplement: Supplementary file 1 [file behavsci-14-01033-s001.zip › behavsci-3243873-supplementary.pdf]

Table S1. Descriptive statistics of the 19 items of COVID-19-PTSD in infected group.

**Item**	Mean	Standard Deviation	Skewness	Kurtosis	Item-total correlation
1. Having repeated, disturbing and unwanted thoughts related to this stressful experience.	1.65	1.19	1.17	-0.94	0.83
2. Having repeated and disturbing dreams related to this stressful experience.	1.39	1.14	0.33	-0.84	0.84
3. Feeling very upset.	1.48	1.16	0.25	-0.95	0.86
4. Having strong physical reactions thinking about this stressful experience (e.g., pounding heart,	1.45	1.17	0.30	-0.91	0.85
5. Try to avoid thoughts and feelings related to this stressful experience.	1.58	1.18	0.21	-0.94	0.84
6. (Having difficulty thinking about aspects other than this stressful situation.	1.46	1.13	2.62	-0.82	0.85
7. Having strong negative beliefs about yourself, others or the world (e.g., having thoughts like: I'm sick, someone dear to me is getting sick, the world has become dangerous.	1.50	1.17	0.25	-0.93	0.86
8. Blaming yourself or someone else for failing to adopt appropriate behaviors to the situation (eg., I went pub, restaurant, etc.	1.40	1.17	0.33	-0.93	0.85
9. Having strong negative feelings like fear, horror, anger, guilt or shame.	1.40	1.19	0.36	-0.96	0.88
10. Experience loss of interest in the activities you liked.	1.40	1.15	0.34	-0.87	0.86
11. Feeling distant from other people.	1.39	1.18	0.34	-0.99	0.85
12. Having difficulty to feel positive feelings (e.g., being unable to feel happiness or positive affects for people close to you.	1.42	1.18	0.32	-0.96	0.88
13. Having irritable behavior, outbursts of anger or aggressive actions.	1.36	1.20	0.42	-0.91	0.86
14. Taking too many risks or doing things that could put you at risk.	1.29	1.17	0.43	-0.89	0.86
15. Being hypervigilant over the current condition.	1.39	1.17	0.33	-0.95	0.87
16. To feel to be nervous or easily frightened.	1.39	1.17	0.36	-0.89	0.87
17. To have trouble concentrating.	1.55	1.19	0.25	-0.94	0.87
18. To have difficulty in falling asleep.	1.44	1.17	0.34	-0.84	0.87
19. To have a disturbed sleep.	1.52	1.19	0.25	-0.94	0.84

Note: All item coefficients were significant at the alpha 0.01 level.

Table S2. Descriptive statistics of the 19 items of COVID-19-PTSD in uninfected group.

**Item**	M	Standard Deviation	Skewness	Kurtosis	Item-total correlation
1. Having repeated, disturbing and unwanted thoughts related to this stressful experience.	1.77	1.33	0.04	-1.22	0.86
2. Having repeated and disturbing dreams related to this stressful experience.	1.53	1.22	0.21	-0.99	0.87
3. Feeling very upset.	1.62	1.27	0.12	-1.16	0.89
4. Having strong physical reactions thinking about this stressful experience (e.g., pounding heart,	1.59	1.28	0.17	-1.14	0.89
5. Try to avoid thoughts and feelings related to this stressful experience.	1.69	1.28	0.12	-1.11	0.88
6. (Having difficulty thinking about aspects other than this stressful situation.	1.59	1.22	0.16	-1.03	0.88
7. Having strong negative beliefs about yourself, others or the world (e.g., having thoughts like: I'm sick, someone dear to me is getting sick, the world has become dangerous.	1.64	1.26	0.12	-1.12	0.90
8. Blaming yourself or someone else for failing to adopt appropriate behaviors to the situation (eg., I went pub, restaurant, etc.	1.57	1.26	0.23	-1.06	0.89
9. Having strong negative feelings like fear, horror, anger, guilt or shame.	1.60	1.27	0.18	-1.13	0.91
10. Experience loss of interest in the activities you liked.	1.53	1.24	0.25	-1.02	0.90
11. Feeling distant from other people.	1.59	1.26	0.19	-1.09	0.90
12. Having difficulty to feel positive feelings (e.g., being unable to feel happiness or positive affects for people close to you.	1.59	1.25	0.16	-1.10	0.90
13. Having irritable behavior, outbursts of anger or aggressive actions.	1.57	1.29	0.21	-1.14	0.89
14. Taking too many risks or doing things that could put you at risk.	1.53	1.24	0.23	-1.07	0.90
15. Being hypervigilant over the current condition.	1.58	1.26	0.18	-1.13	0.90
16. To feel to be nervous or easily frightened.	1.56	1.25	0.22	-1.06	0.90
17. To have trouble concentrating.	1.61	1.29	0.20	-1.13	0.88
18. To have difficulty in falling asleep.	1.50	1.22	0.28	-1.00	0.87
19. To have a disturbed sleep.	1.55	1.26	0.23	-1.10	0.87

Note: All item coefficients were significant at the alpha 0.01 level.

Table S3. Comparison of the scores of items in the high and low groups of COVID-19 PTSD among infected group.

**Item**	*t*	*p*
1. Having repeated, disturbing and unwanted thoughts related to this stressful experience.	-78.75	<0.001
2. Having repeated and disturbing dreams related to this stressful experience.	-79.91	<0.001
3. Feeling very upset.	-90.20	<0.001
4. Having strong physical reactions thinking about this stressful experience (e.g., pounding heart, difficulty breathing.	-81.93	<0.001
5. Try to avoid thoughts and feelings related to this stressful experience.	-82.27	<0.001
6. (Having difficulty thinking about aspects other than this stressful situation.	-81.62	<0.001
7. Having strong negative beliefs about yourself, others or the world (e.g., having thoughts like: I'm sick, someone dear to me is getting sick, the world has become dangerous.	-89.70	<0.001
8. Blaming yourself or someone else for failing to adopt appropriate behaviors to the situation (eg., I went pub, restaurant, etc.	-87.83	<0.001
9. Having strong negative feelings like fear, horror, anger, guilt or shame.	-98.37	<0.001
10. Experience loss of interest in the activities you liked.	-87.49	<0.001
11. Feeling distant from other people.	-92.41	<0.001
12. Having difficulty to feel positive feelings (e.g., being unable to feel happiness or positive affects for people close to you.	-92.31	<0.001
13. Having irritable behavior, outbursts of anger or aggressive actions.	-93.61	<0.001
14. Taking too many risks or doing things that could put you at risk.	-91.99	<0.001
15. Being hypervigilant over the current condition.	-93.62	<0.001
16. To feel to be nervous or easily frightened.	-92.46	<0.001
17. To have trouble concentrating.	-82.77	<0.001
18. To have difficulty in falling asleep.	-69.31	<0.001
19. To have a disturbed sleep.	-72.75	<0.001

Note: All item coefficients were significant at the alpha 0.01 level.

Table S4. Comparison of the scores of items in the high and low groups of COVID-19 PTSD among uninfected group.

**Item**	*t*	*p*
1. Having repeated, disturbing and unwanted thoughts related to this stressful experience.	-76.66	<0.001
2. Having repeated and disturbing dreams related to this stressful experience.	-74.12	<0.001
3. Feeling very upset.	-87.41	<0.001
4. Having strong physical reactions thinking about this stressful experience (e.g., pounding heart, difficulty breathing.	-85.87	<0.001
5. Try to avoid thoughts and feelings related to this stressful experience.	-76.35	<0.001
6. (Having difficulty thinking about aspects other than this stressful situation.	-76.14	<0.001
7. Having strong negative beliefs about yourself, others or the world (e.g., having thoughts like: I'm sick, someone dear to me is getting sick, the world has become dangerous.	-89.86	<0.001
8. Blaming yourself or someone else for failing to adopt appropriate behaviors to the situation (eg., I went pub, restaurant, etc.	-83.84	<0.001
9. Having strong negative feelings like fear, horror, anger, guilt or shame.	-96.08	<0.001
10. Experience loss of interest in the activities you liked.	-85.26	<0.001
11. Feeling distant from other people.	-88.71	<0.001
12. Having difficulty to feel positive feelings (e.g., being unable to feel happiness or positive affects for people close to you.	-84.78	<0.001
13. Having irritable behavior, outbursts of anger or aggressive actions.	-85.71	<0.001
14. Taking too many risks or doing things that could put you at risk.	-84.97	<0.001
15. Being hypervigilant over the current condition.	-91.19	<0.001
16. To feel to be nervous or easily frightened.	-86.20	<0.001
17. To have trouble concentrating.	-81.72	<0.001
18. To have difficulty in falling asleep.	-74.13	<0.001
19. To have a disturbed sleep.	-78.23	<0.001

Note: All item coefficients were significant at the alpha 0.01 level.

Table S5. Specific information on COVID-19 infection and recovery of infected groups (n = 3617).

Characteristic	N	%	COVID-19 PTSS (M±SD)	F	*P*
**Infection situation**				62.86	**<0.001**
recovered (PCR/antigen test negative)	3081	85.2%	26.07±18.86		
symptoms improved (PCR/antigen	421	11.6%	33.97±17.83		
currently infected	115	3.2%	40.57±17.06		
**Time of COVID-19 infection**				129.66	**<0.001**
Within the last three days	158	4.4%	37.12±18.10		
Within the last week	523	14.5%	38.32±16.96		
Within the last two weeks	1625	44.9%	27.94±18.58		
At other time	1311	36.2%	21.34±17.62		
**Days from infection to recovery**				8.30	**<0.001**
0-14 days	3257	90%	26.86±18.77		
15-30 days	294	8.2%	31.44±19.27		
More than 30 days	18	0.5%	30.72±19.04		
**Severity of COVID-19**			27.26±18.85	67.07	**<0.001**
Asymptomatic[a]	479	13.2%	26.22±20.26		
Mild symptoms[b]	2553	70.6%	25.16±18.30		
Moderate symptoms[c]	454	12.6%	37.06±17.07		
Severe symptoms[d]	105	2.9%	42.33±12.51		
Critical condition[e]	26	0.7%	47.23±15.15		

Note: [a]:no fatigue, fever, cough, or other evident symptoms are present, though there may be a loss of smell/taste; [b]: characterized by symptoms such as cough, fever, muscle ache, etc., but without pneumonia; [c]: referring to non-severe pneumonia requiring various drug treatments but no additional oxygen therapy; [d]: involving serious pneumonia necessitating extra oxygen therapy, possibly even intravenous injections; [e]: life-threatening, including the need for invasive ventilation/respiratory support, intravenous injections, sepsis, cardiac disease, stroke, embolism, acute respiratory distress syndrome (ARDS).

Table S6. Comparison of emotional symptoms among uninfected group and infected group.

Characteristic	UG (n=2336)	IG (n=3617)
Anxiety level	4.76±2.79	4.06±2.69
Depression level	4.40±2.83	3.58±2.74
Stress level	4.70±2.74	4.08±2.69
Loneliness level	4.52±2.77	3.74±2.78

UG: Uninfected group; IG: Infected group; The infected group assessed their level of worry, depression and anxiety after contracting COVID-19. The uninfected group rated their level of worry, depression and anxiety about COVID-19 infection.

Table S7. Results of structural model for full sample and subsamples.

Model Paths	Full Sample			UG			IG		
	β	SE	CR	*β*	SE	CR	*β*	SE	CR
COVID-19 PTSS <--- Chronic fatigue	0.02	0.04	1.776	0.11***	0.04	5.742	0.01	0.05	0.724
COVID-19 PTSS <--- Insomnia symptoms	0.37***	0.09	24.504	0.33***	0.16	14.076	0.41***	0.10	21.071
Insomnia symptoms <--- Chronic fatigue	0.44***	0.01	30.357	0.34***	0.01	14.668	0.46***	0.01	24.976

***$p < 0.001$; UG: Uninfected group; IG: Infected group; *β*, standardized coefficient; SE: standard error; CR: critical ratio; COVID-19 PTSS: Post-traumatic stress symptoms related to the COVID-19 pandemic; Adjusted for age, gender, drinking, smoking, and regular physical activity.

Table S8. Direct and indirect effects and 95% confidence intervals (CI).

Model Pathways	Full Sample			UG			IG		
	Estimated	95%CI		Estimated	95%CI		Estimated	95%CI	
		Lower	Upper		Lower	Upper		Lower	Upper
Direct effect									
COVID-19 PTSS <--- Chronic fatigue	0.07	-0.212	0.149	0.23	0.139	0.320	0.03	-0.068	0.40
Indirect effect									
COVID-19 PTSS <--- Insomnia symptoms <--- Chronic fatigue	0.43	0.387	0.475	0.33	0.274	0.399	0.48	0.426	0.545

UG: uninfected group; IG: infected group; CI: confidence intervals.
